# Simultaneous occurrence of a rare lymphoepithelial cyst and squamous cell carcinoma in the oral cavity

**DOI:** 10.1590/S1808-86942011000200022

**Published:** 2015-10-19

**Authors:** Fabio Wildson Gurgel Costa, Karuza Maria Alves Pereira, Thales Salles Angelim Viana, Roberta Barroso Cavalcante, Alexandre Simões Nogueira

**Affiliations:** 1 MSc in Dentistry, Assistant Professor of Dentistry - Universidade Federal do Ceará; 2 MSc in Oral Pathology - UFRN; PhD student - Graduate Program in Dentistry - UFRN, Assistant Professor of Dentistry - UFC Campus Sobral; 3 Dentistry student - UFC Campus Sobral, 4th Year Student of Dentistry - UFC Campus Sobral; 4 PhD in Oral Pathology - UFRN, Professor of Dentistry - UNIFOR; 5 MSc in CTBMF - UFPE; PhD student - Graduate Program in Dentistry - UFC Campus Sobral. Universidade Federal do Ceará Campus Sobral

**Keywords:** squamous cell, carcinoma, nonodontogenic cysts, mouth

## INTRODUCTION

Lymphoepithelial cysts are rare in the oral cavity. Located mainly on the mouth floor^1^, ^2^, ^3^. Contrary to that, the epidermoid carcinoma is a relatively uncommon malignant neoplasia in the oral cavity, which diagnosis is usually made at advanced stages^4^. Scarce reports mention the coexistence of these lesions with entities such as the epidermoid cyst^3^ and geographic tongue^2^. Nonetheless, so far, the simultaneous occurrence of both lesions has not been reported in the oral cavity. Thus, the goal of the present study was to report on an interesting and extremely uncommon case of these lesions in adjacent anatomical sites.

## CASE PRESENTATION

**Figure 1:**
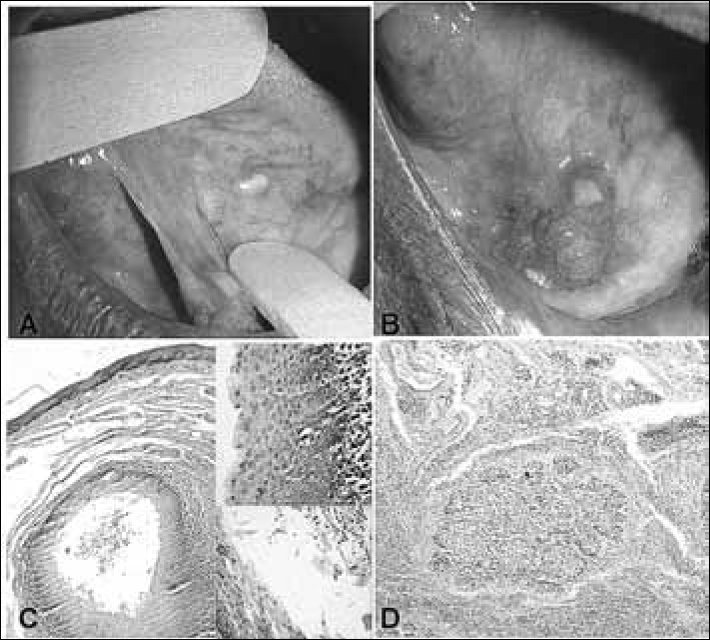
A) Papule-yellowish lesion on the tongue belly surface; B) nodule-ulcerated lesion in the anterior region of the mouth floor; C) Microphotography showing a histology pattern matching those of a lymphoepithelial cyst (HE, x100); D) Microphotography showing nests of malignant cells invading the connective tissue, characterizing the epidermoid carcinoma (HE, x100)

A male, 71-year old patient was referred to us because of a painless intraoral lesion, perceived 6 months ago, with a granulomatous and ulcerated surface, firm to the touch, approximate size of 2cm and located in the anterior region of the mouth floor. Upon oral examination, we noticed another yellowish papular lesion measuring about 3mm; thus, the diagnostic hypotheses were of epidermoid carcinoma and granular cell tumor, respectively. We biopsied the lesion suggestive of malignant neoplasia and excised the other. After the pathology exam, the diagnosis was of epidermoid carcinoma (a malignant neoplasia characterized by proliferation invading the underlying connective tissue, with the cells showing cellular and nuclear polymorphism, and also cell hyperchromatism and mitotic figures) and lymphoepithelial cyst (a pathological cavity coated by keratinized stratified squamous epithelium, showing a lymphoid center in the juxta-epithelial, respectively (Fig.1). the patient was then referred to oncologic treatment, and today the patient is without significant changes.

## DISCUSSION

The lymphoepithelial cyst is a benign, very uncommon lesion in the oral cavity. When intraoral, it affects mainly the mouth floor (65.3%), followed by the posterior region of the tongue (13.7%). Nonetheless, in the present case, the lesion involved the tongue belly. Its development happens on the oral lymphoid tissue normally surrounded by areas of keratin from the coating epithelium^2^, ^3^. Its etiology is still not well described in the literature, local trauma is one hypothesis, although our patient did not report it. The epidermoid carcinoma represents about 95% of the malignant lesions which affect the oral cavity with associated etiology, especially smoking and alcoholism^4^, these data corroborate the present paper.

Pereira et al. (2009)^2^ reported its occurence among lymphoepithelial cysts and benign migratory glossitis, suggesting a possible immune background for this disease. Epivatianos et al. (2005)^3^ reported the association between lymphoepithelial cysts and epidermoid carcinoma on the mouth floor. Using Pubmed data we have so far, there are no reports of the coexistence of lymphoepithelial cysts and epidermoid carcinoma. We believe that such coexistence must not have the same etiology, but rather a very particular one. Thus, our approach followed the guidelines of each lesion, respecting their biological behaviors.

## FINAL REMARKS

We stress the importance of a careful clinical exam for asymptomatic and small lesions, regardless of ulceration and ease of recognition, as well as the role of professionals who work with the oral cavity, namely dentists and ENTs, in the early diagnosis of malignant lesions.
